# Deep learning-based automated segmentation and quantification of the dural sac cross-sectional area in lumbar spine MRI

**DOI:** 10.3389/fradi.2025.1503625

**Published:** 2025-03-25

**Authors:** George Ghobrial, Christian Roth

**Affiliations:** ^1^ Clinic for Diagnostic and Interventional Radiology and Neuroradiology, Klinikum Bremerhaven Reinkenheide, Bremerhaven, Germany; ^2^ Clinic for Diagnostic and Interventional Neuroradiology, Klinikum Bremen-Mitte/Bremen-Ost, Bremen, Germany

**Keywords:** deep learning, lumbar spine MRI, dural sac cross-sectional area (DSCA), medical image segmentation, MultiResUNet, automated diagnosis, spinal canal stenosis, radiological assessment

## Abstract

**Introduction:**

Lumbar spine magnetic resonance imaging (MRI) plays a critical role in diagnosing and planning treatment for spinal conditions such as degenerative disc disease, spinal canal stenosis, and disc herniation. Measuring the cross-sectional area of the dural sac (DSCA) is a key factor in evaluating the severity of spinal canal narrowing. Traditionally, radiologists perform this measurement manually, which is both time-consuming and susceptible to errors. Advances in deep learning, particularly convolutional neural networks (CNNs) like the U-Net architecture, have demonstrated significant potential in the analysis of medical images. This study evaluates the efficacy of deep learning models for automating DSCA measurements in lumbar spine MRIs to enhance diagnostic precision and alleviate the workload of radiologists.

**Methods:**

For algorithm development and assessment, we utilized two extensive, anonymized online datasets: the “Lumbar Spine MRI Dataset” and the SPIDER-MRI dataset. The combined dataset comprised 683 lumbar spine MRI scans for training and testing, with an additional 50 scans reserved for external validation. We implemented and assessed three deep learning models—U-Net, Attention U-Net, and MultiResUNet—using 5-fold cross-validation. The models were trained on T1-weighted axial MRI images and evaluated on metrics such as accuracy, precision, recall, F1-score, and mean absolute error (MAE).

**Results:**

All models exhibited a high correlation between predicted and actual DSCA values. The MultiResUNet model achieved superior results, with a Pearson correlation coefficient of 0.9917 and an MAE of 23.7032 mm^2^ on the primary dataset. This high precision and reliability were consistent in external validation, where the MultiResUNet model attained an accuracy of 99.95%, a recall of 0.9989, and an F1-score of 0.9393. Bland-Altman analysis revealed that most discrepancies between predicted and actual DSCA values fell within the limits of agreement, further affirming the robustness of these models.

**Discussion:**

This study demonstrates that deep learning models, particularly MultiResUNet, offer high accuracy and reliability in the automated segmentation and calculation of DSCA in lumbar spine MRIs. These models hold significant potential for improving diagnostic accuracy and reducing the workload of radiologists. Despite some limitations, such as the restricted dataset size and reliance on T1-weighted images, this study provides valuable insights into the application of deep learning in medical imaging. Future research should include larger, more diverse datasets and additional image weightings to further validate and enhance the generalizability and clinical utility of these models.

## 1 Introduction

Low back pain (LBP) is a pervasive condition globally, significantly impacting individuals and healthcare systems. Classified into axial lumbosacral pain, radicular pain, and referred pain (1, 2), it affects the lumbar spine and sacrum, manifests along dermatomal patterns, or spreads to non-dermatomal areas. LBP is a leading cause of disability and medical consultation (3, 4), with an annual prevalence of 10%–30% and a lifetime prevalence of 65%–80% (5). It is categorized into acute (<6 weeks), subacute (6–12 weeks), and chronic (>12 weeks) phases (3, 6). Despite many cases resolving within six weeks, 10%–40% persist, requiring differentiated management strategies (3).

Initial management of acute and subacute LBP focuses on ruling out serious underlying conditions (“red flags”) and encouraging activity (2, 3, 5). Chronic LBP, affecting a significant proportion of patients, necessitates a multidisciplinary approach integrating medical, psychological, and physical therapies (7). Imaging, particularly MRI, plays a crucial role in diagnosing and planning treatment for various spinal pathologies such as degenerative disc diseases, spinal stenosis, and herniated discs (8). MRI offers superior soft tissue contrast without ionizing radiation, making it the preferred modality for detailed spinal assessments (9).

Evaluating the severity of spinal canal stenosis and related symptoms relies heavily on the measurement of the cross-sectional area of the dural sac (DSCA) (10). Historically, this measurement has been performed manually by radiologists, which is not only time-consuming but also subject to variability and errors (11). Consistent and accurate DSCA measurement is essential for effective diagnosis and treatment planning, highlighting the need for automated solutions. Deep learning (DL), especially Convolutional Neural Networks (CNNs), has demonstrated substantial potential in the analysis of medical images, including tasks like segmentation, classification, and detection (12). The U-Net model, a widely recognized CNN architecture, has achieved success in numerous biomedical segmentation tasks, including the segmentation of cells, organs, and tumors (13). The 3D U-Net architecture has been successfully adapted for volumetric medical image segmentation, demonstrating state-of-the-art performance in various applications, including brain tumor and spinal structure segmentation, with superior Dice similarity coefficients and enhanced segmentation accuracy (14–16). Studies have highlighted the importance of architectural modifications, such as dilated convolutions and advanced decoder designs, in addressing the unique challenges of 3D imaging (15, 17). Furthermore, efforts like the “nnU-Net” framework emphasize rigorous validation and adaptability of U-Net models to diverse datasets, achieving consistent results in clinical applications (15, 17). Automated measurement systems based on deep learning hold transformative potential beyond lumbar spine cases, extending their applicability to various clinical domains. For instance, these methods can streamline the analysis of brain, cardiac, and abdominal imaging, where precise and consistent measurements are critical for diagnosing complex conditions like tumors, arrhythmias, and organ-specific pathologies (18, 19). Despite the potential, the application of DL models specifically for automated DSCA measurement in lumbar spine MRIs remains underexplored. Manual DSCA measurement not only demands considerable time but also exhibits inter-radiologist variability, affecting diagnostic accuracy (20). An automated, DL-based approach could overcome these limitations by providing consistent, objective measurements, thereby improving diagnostic accuracy and aiding treatment planning. Furthermore, such a method could alleviate the workload of radiologists, enabling more efficient use of resources.

This research focuses on developing and evaluating DL algorithms for the automated detection and quantification of DSCA in axial T1-weighted lumbar spine MRI scans. By utilizing publicly accessible MRI datasets, this study aims to train and validate these models to determine their effectiveness in automating DSCA measurement. The primary objective is to advance the research on DL applications in medical imaging, thereby enhancing efficiency and accuracy in the assessment of spinal conditions and improving patient outcomes through more informed treatment planning. By automating DSCA measurement, this research seeks to evaluate a reliable tool that can consistently deliver accurate results, supporting radiologists and clinicians in diagnosing and treating spinal conditions. This advancement not only has the potential to streamline clinical workflows but also to standardize measurements, reducing variability and improving overall patient care. The insights gained from this study could pave the way for the development of DL models for other clinically relevant features in spine MRI, promoting broader applications in medical imaging and beyond.

## 2 Material and methods

### 2.1 Study design and ethical considerations

This study utilized two large, publicly available datasets from other universities to develop the algorithm (21–23). Both datasets were anonymized and made available for research purposes. The “Lumbar Spine MRI Dataset” from Liverpool John Moores University, England, encompasses 515 cases with symptomatic back pain and various demographic characteristics. All procedures conformed to the ethical standards of the United Kingdom and the Kingdom of Jordan and complied with the Declaration of Helsinki of 1964 and its later amendments. Data were collected between September 2015 and July 2016 from patients who visited the hospital with corresponding symptoms. Written informed consent was obtained from each patient before data collection. Personal identifiers were removed to anonymize the data, including patient names, birthdates, and visit dates. The MRI scans included axial views of the lower three lumbar vertebrae and intervertebral discs, with most image slices having a resolution of 320 × 320 pixels at 12 bits per pixel. T1-weighted MRI images from this dataset were used.

The SPIDER MRI dataset from Radboud University Medical Center Nijmegen included 218 patients. In total, 447 MRI series of the lumbar spine were retrospectively collected from patients with a history of lower back pain. Institutional review board approval was gained from the Radboud University Medical Center (approval number: 2016–2275). Data were collected from four different hospitals in the Netherlands, including a university hospital, two regional hospitals, and an orthopedic hospital, between January 2019 and March 2022. Axial T1 sequences (voxel size: 3.30 × 0.59 × 0.59 mm) from this dataset were included. A subset of 50 patients from the combined datasets was used as an external validation set to evaluate the models further, ensuring that these data were not used during model training. Thus, a total of 683 data points were used for training and testing, with an additional 50 data points for external validation.

### 2.2 Study population and data sources

**Figure 1:**
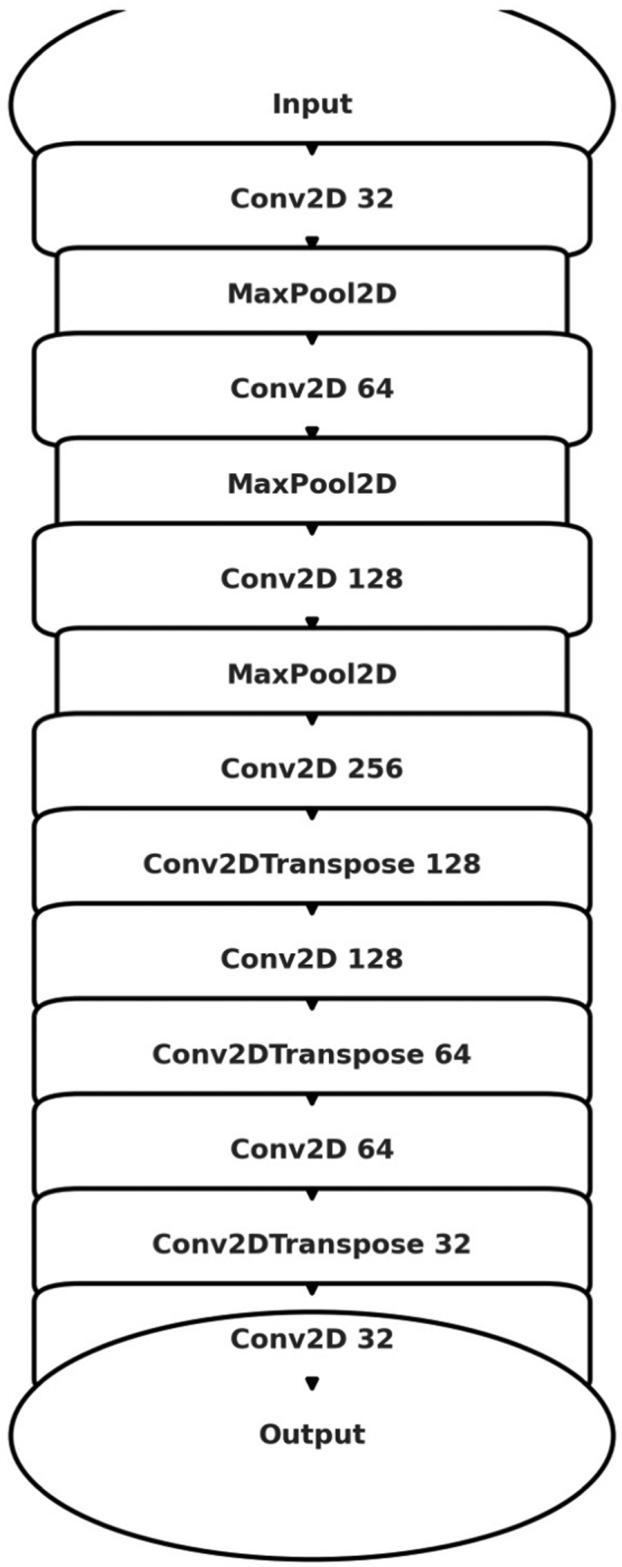
Diagram illustrating the 5-fold cross-validation process applied to a dataset of 683 lumbar spine MRI scans. The dataset is divided into five equal folds, each serving as the test set once while the remaining four folds serve as the training set. This process is repeated five times, ensuring each fold is used as the test set once. Additionally, an external validation set consisting of 50 independent lumbar spine MRI scans is used to further assess the model's performance. The use of cross-validation and an independent external dataset ensures robust model evaluation.

The dataset used comprised T1-weighted axial MRI scans, along with manually segmented data to serve as the Ground Truth. The images were standardized to a resolution of 320 × 320 pixels and normalized by scaling each pixel's intensity by 255.0. To binarize the segmented slices, pixels with an intensity value of 150/255 were set to 1.0, while all other pixels were set to 0.0. We standardized the MRI scans to a uniform resolution and normalized their intensities, ensuring a consistent intensity range that facilitates reliable model convergence and performance comparison. To produce clear and consistent binary segmentation masks, pixels above an intensity threshold of 150/255 were set to 1.0, distinctly separating the dural sac from surrounding tissues. To ensure a comprehensive evaluation of the models’ performance across various subsets of data, a 5-fold cross-validation approach was employed (Figure 1). Employing a 5-fold cross-validation strategy allowed each dataset partition to serve as a validation set once, reducing variance in model evaluation and improving generalizability. This rigorous approach provided a more stable and comprehensive assessment of the model's robustness, ensuring that the reported performance metrics are both reliable and applicable to diverse clinical scenarios. Each of the three deep learning architectures—U-Net, Attention U-Net, and MultiResUNet—underwent training and validation five times, with each fold serving once as the validation set, while the other four folds constituted the training set.

### 2.3 Segmentation

**Figure 2:**
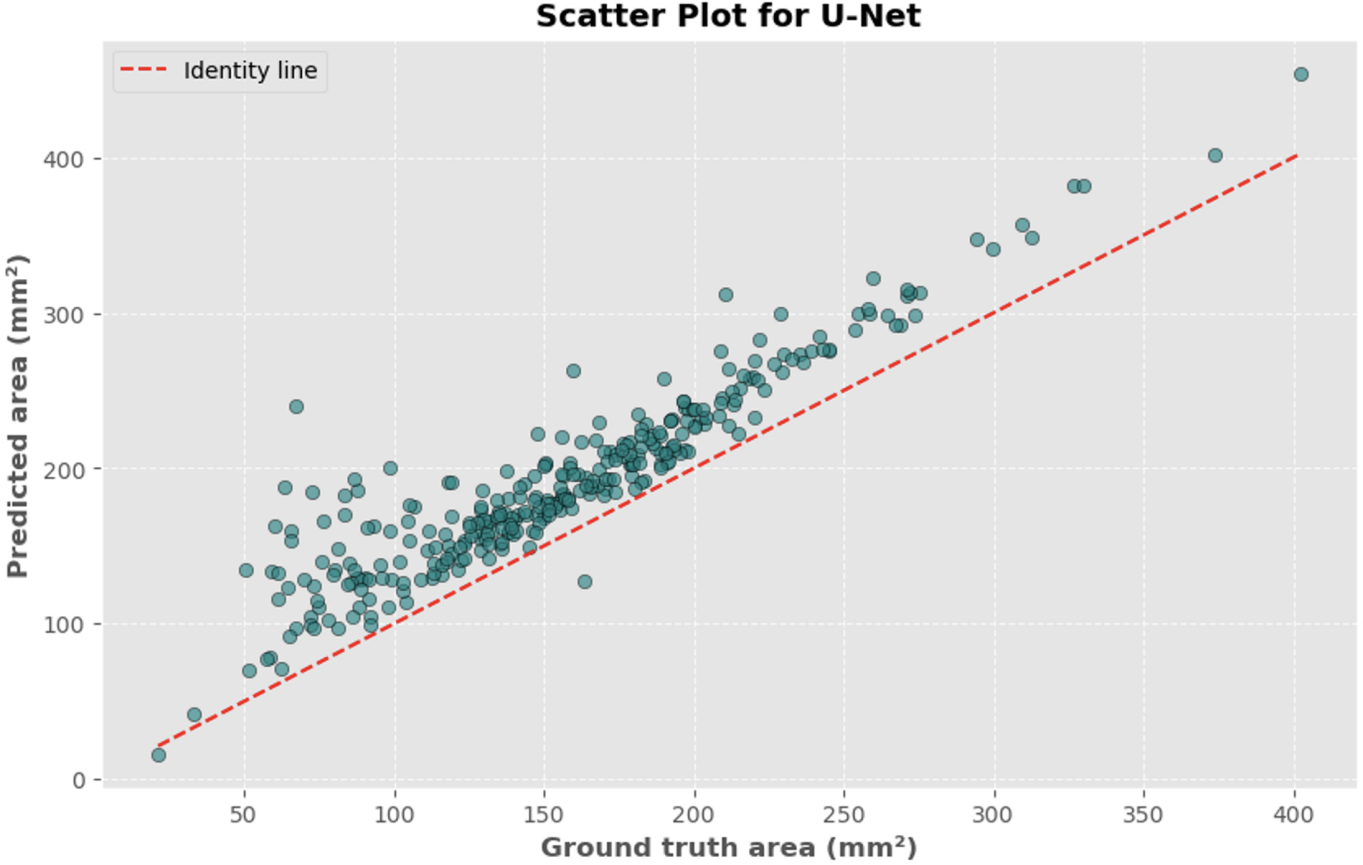
Representation of the segmentation process using 3D slicer (version 5.2.2) (24). An intensity histogram is used to pre-mark a part of the DSCA on a representative slice, and an AI-based automated algorithm fills in adjacent areas of the DSCA. This allows for precise segmentation of the region.

**Figure 3:**
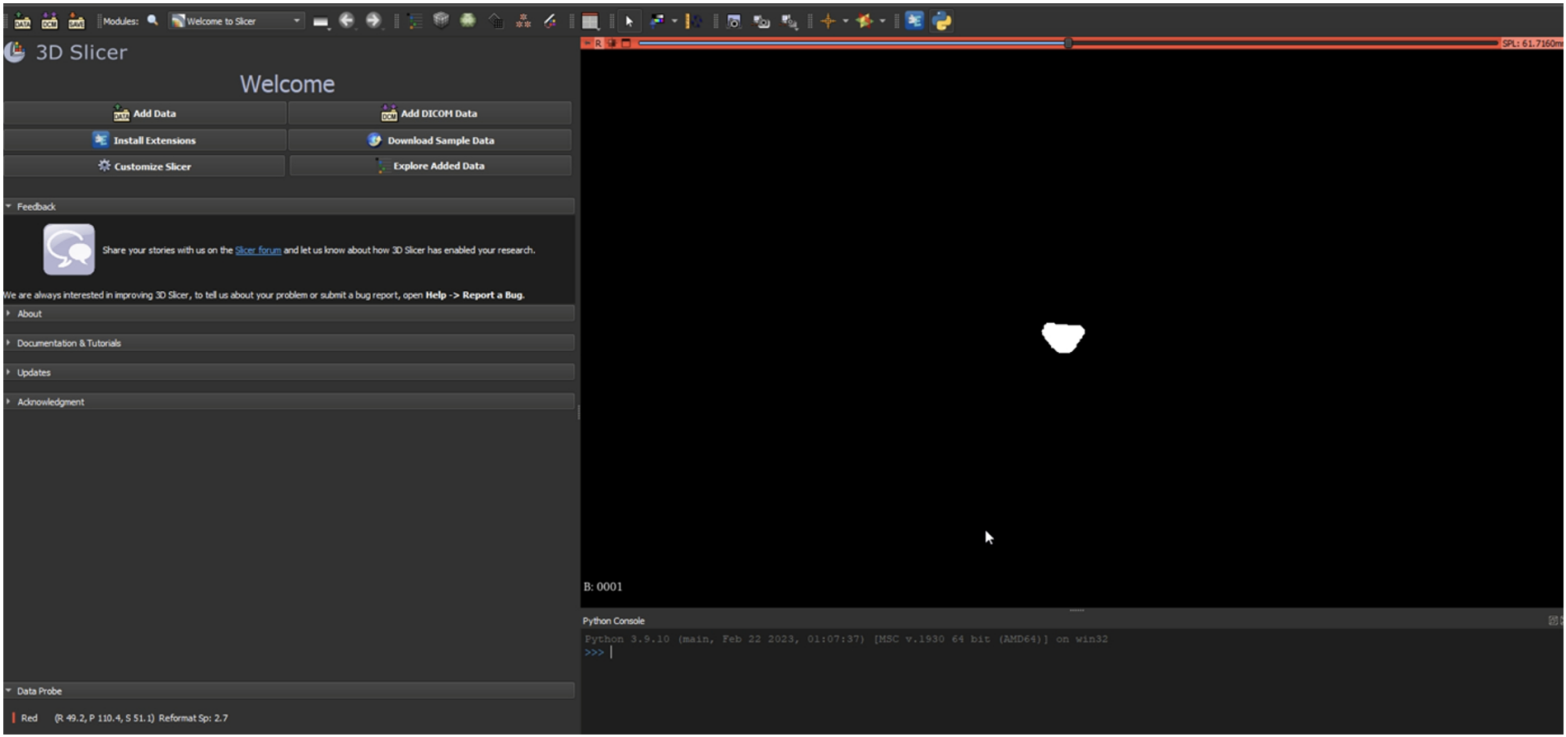
Illustration of a segmentation mask from a segmentation process. This mask is used as the “Ground-Truth” for the algorithm to learn the actual DSCA area and compare it with the algorithm's prediction.

Ground truth for training and testing machine learning algorithms for image segmentation was derived from annotated images marking the dural sac (Region of Interest, ROI). The manual delineation and refinement of the segmentation masks were performed by the lead author (GG), a senior physician radiologist with over 15 years of clinical experience in diagnostic radiology and neuroradiology, ensuring accurate and reliable annotations. The annotation process involved segmenting the dural sac on the slices using the 3D Slicer software (version 5.2.2) (24). For segmentation, the most representative axial slices of the final three segments were chosen, specifically those closest to the median height of the intervertebral disc. Lumbar T1-weighted MRI DICOM images were processed and imported into the 3D Slicer software (24). A combination of manual and semi-automatic techniques was employed for segmenting the dural sac. Initially, the central portions of the dural sac were manually delineated, and local intensity histograms were utilized to establish thresholds (Figure 2). These thresholds facilitated the volumetric segmentation of the dural sac, extending to adjacent slices, with subsequent manual refinements. Each patient's segmentation encompassed 3–8 slices of the lumbar region, culminating in a final segmentation mask that served as the ground truth (Figure 3).

### 2.4 Development of the AI-based algorithm

**Figure 4:**
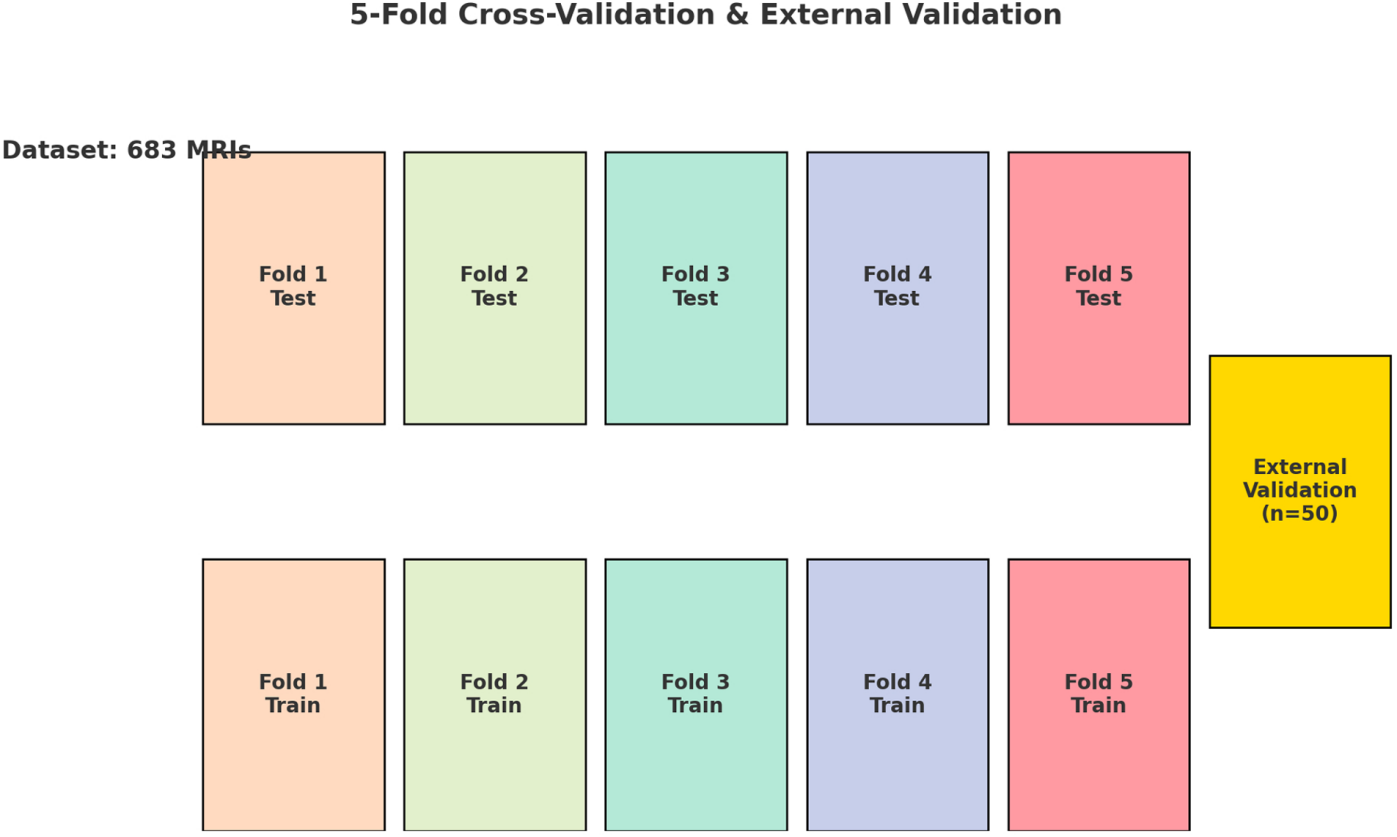
Schematic illustration of the U-Net architecture utilized for image segmentation. The model exhibits the characteristic U-shaped encoder-decoder structure with symmetric contracting (encoding) and expansive (decoding) paths interconnected through skip connections. The encoding path employs convolutional layers (Conv2D) followed by max-pooling layers (MaxPooling2D) to progressively reduce spatial dimensions while increasing feature channel depth. Conversely, the decoding path applies transposed convolutional layers (Conv2DTranspose) coupled with skip connections from corresponding encoding layers to reconstruct high-resolution segmentation masks.

Three distinct architectures were implemented for the automatic identification and computation of the dural sac cross-sectional area. These architectures included the basic U-Net model (Figure 4), an enhanced Attention U-Net variant, and a MultiResUNet model. Supplementary Table S1 provides an overview, contrasting the key architectural and functional differences between U-Net, Attention U-Net, and MultiResUNet. The models were built using the Adam optimizer and a specially designed weighted binary cross-entropy loss function. Following a manual grid search, the weights for the positive and negative classes were set at 20.0 and 1.0, respectively. The basic U-Net model consisted of several layers, including convolutional layers, max-pooling layers, and up-sampling layers, all activated with ReLU functions. The Attention U-Net variant introduced attention gates to emphasize important features during training, thereby enhancing the model's focus on specific image regions. The MultiResUNet model incorporated multi-resolution analysis, featuring a multi-resolution block with three convolutional layers and ResPaths to capture detailed image information. Training for each model was conducted with a batch size of 8 across 20 epochs. A ModelCheckpoint callback was employed to save the optimal model based on validation loss.

The performance of the trained models was assessed by calculating the mean absolute error (MAE) between the predicted and actual areas. To illustrate model performance, scatter plots and Bland-Altman plots were generated. For each model, a representative image was selected to showcase the input image, ground truth segmentation, and predicted segmentation, along with the respective cross-sectional areas (in mm^2^). The function calculate_area was utilized to determine the dural sac cross-sectional area from the segmentation mask. This function required two inputs: the binary mask of the segmented image and the pixel size in millimeters (derived from the DICOM volume information). The function computed the total number of pixels in the mask using np.sum and then multiplied this by the square of the pixel size in millimeters to obtain the area in square millimeters, with the final output being the calculated area in mm^2^.

### 2.5 Evaluation of the algorithm and evaluation metrics

To assess the performance of the deep learning models, various statistical metrics were computed. The models evaluated included a standard U-Net, an Attention U-Net, and a MultiResUNet. Performance metrics such as accuracy, precision, recall, and F1-score were used to gain insights into the models’ capabilities. These metrics evaluated the models’ precision in correctly identifying positive instances, their recall in recognizing actual positive instances, overall accuracy, and the harmonic mean of precision and recall (F1-score). Specifically, accuracy was calculated as the ratio of correct predictions (both positive and negative) to the total number of predictions. Precision was defined as the ratio of true positive predictions to the total of true and false positive predictions. Recall was the ratio of true positive predictions to the total of true positive and false negative predictions. The F1-score was calculated as the harmonic mean of precision and recall, providing a balanced assessment of model performance.

Beyond these fundamental metrics, the absolute error in estimating the cross-sectional area of the dural sac in the segmented images was evaluated by comparing the predicted segmentation area with the ground truth. For each model, the mean absolute error (MAE) was computed. The MAE provided a measure of the average magnitude of errors, disregarding their direction. Additionally, the correlation between the actual and predicted cross-sectional areas of the dural sacs was examined using Pearson's correlation coefficient. This coefficient measured the linear relationship between two datasets, with +1 indicating a perfect positive correlation, −1 a perfect negative correlation, and 0 no correlation. Furthermore, a Bland-Altman analysis was conducted to evaluate the agreement between the actual and predicted areas. This involved plotting the differences between the predicted and actual areas against the mean of these values and calculating the mean difference and limits of agreement (mean difference ± 1.96 standard deviations). This statistical approach is widely used in medical studies to compare two measurement methods.

The statistical evaluation provided a comprehensive understanding of the models’ performance in terms of segmentation accuracy, area estimation, and correlation with actual data. This detailed analysis enabled reliable comparisons between the U-Net, Attention U-Net, and MultiResUNet models. An external dataset, not previously encountered by the models, was also used for validation. The dataset underwent the same preprocessing and binarization procedures as the main dataset. Model performance on this external dataset was evaluated using the same metrics. All analyses were conducted using Python version 3.10, employing modules such as TensorFlow, Keras, NumPy, OpenCV, and Matplotlib. All model training and evaluation were conducted on a workstation equipped with an AMD Ryzen 9 5950X 16-Core Processor (Santa Clara, CA, USA) and 64 GB of RAM, ensuring ample computational capacity for handling large MRI datasets and iterative model experimentation. The graphics processing was managed by an NVIDIA GeForce RTX 3090 GPU (Santa Clara, CA, USA), which facilitated efficient parallelization and accelerated training of our deep learning models. Our software environment included Python version 3.10 (64-bit, Wilmington, DE, USA) running on Windows 10 (Redmond, WA, USA).

## 3 Results

### 3.1 Bland-Altman and correlation analysis

**Figure 5:**
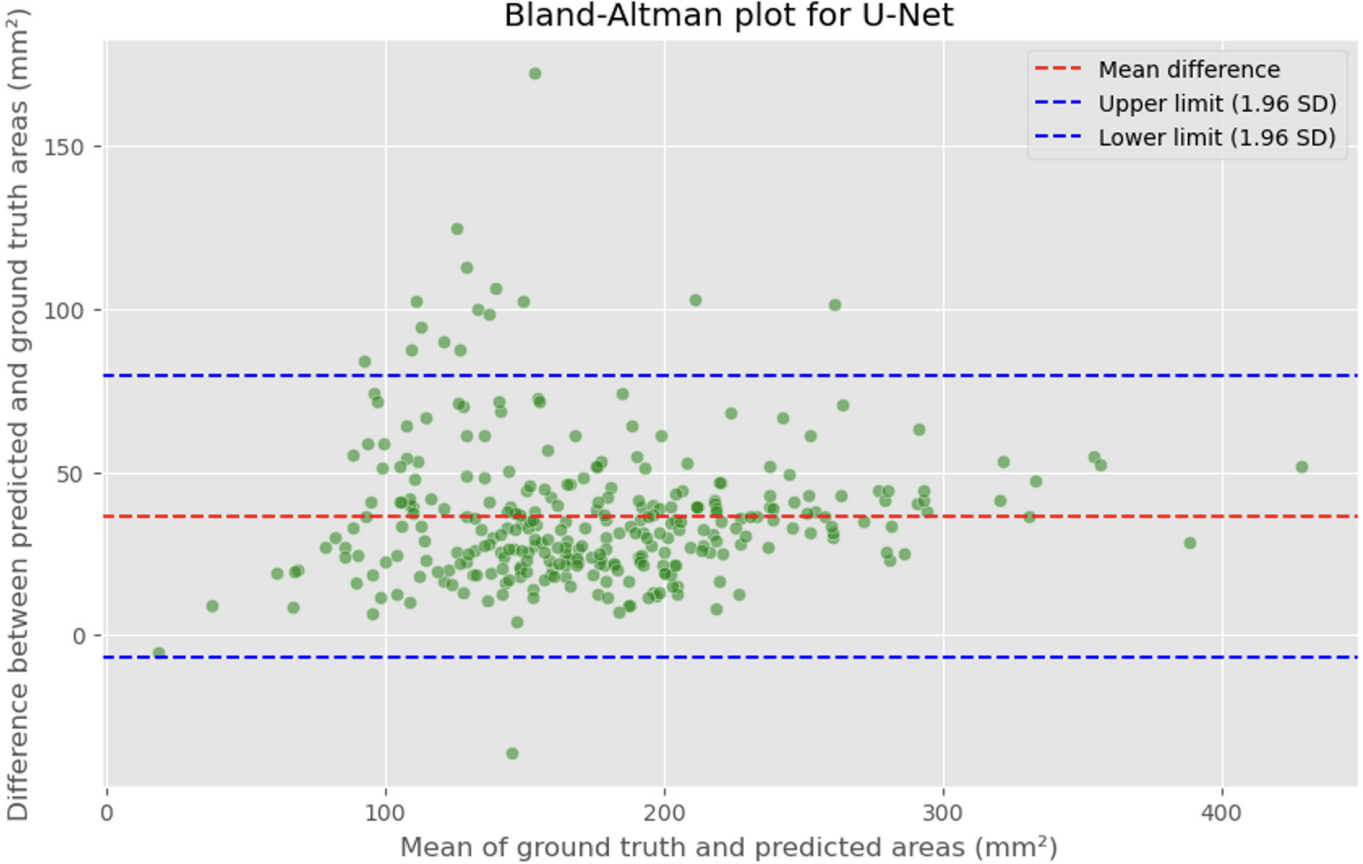
Bland-Altman plot showing the difference between predicted and actual DSCA areas against their mean. The red dashed line represents the mean difference, with values indicated for the U-Net model. The blue dashed lines represent the limits of agreement, calculated as the mean difference ± 1.96 times the standard deviation of the differences. The mean difference is 36.4098 mm^2^, with limits of agreement ranging from −6.8582 to 79.6779 mm^2^.

**Figure 6:**
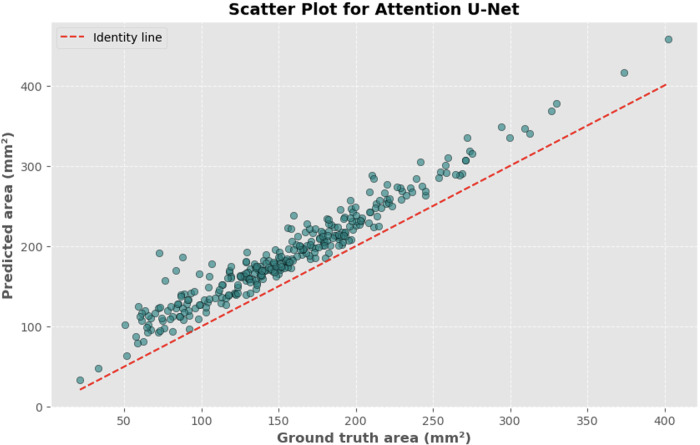
Scatter plot depicting the relationship between actual and predicted DSCA areas. Each point represents a single image from the validation set (identified using the best model from k-fold cross-validation with sklearn.model_selection in Python), showing results for the U-Net model. Pearson's correlation coefficient: 0.9323.

**Figure 7:**
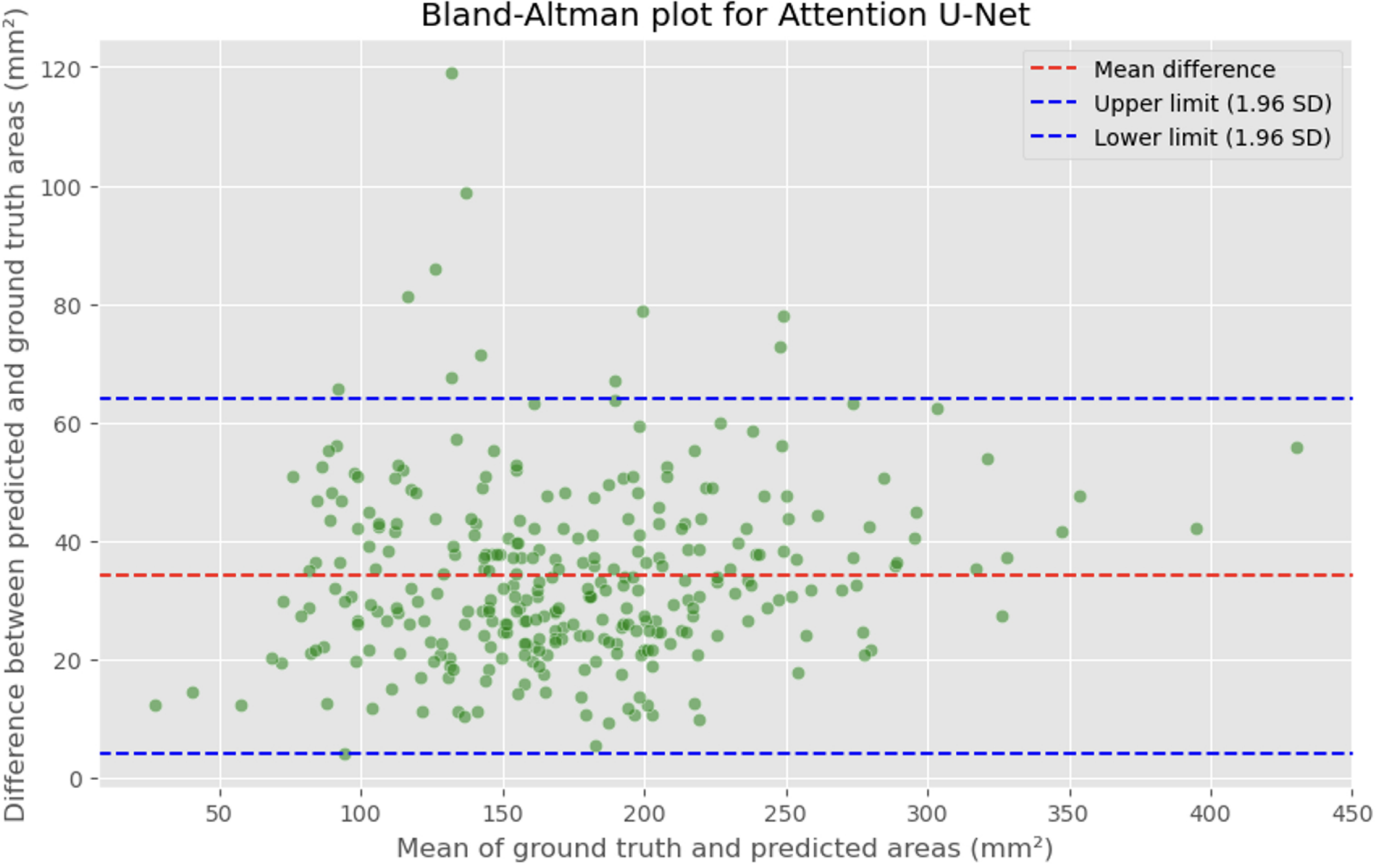
Bland-Altman plot showing the difference between predicted and actual DSCA areas against their mean. The red dashed line represents the mean difference, with values indicated for the Attention U-Net model. The blue dashed lines represent the limits of agreement, calculated as the mean difference ± 1.96 times the standard deviation of the differences. The mean difference is 34.1307 mm^2^, with limits of agreement ranging from 4.2782 to 63.9832 mm^2^.

**Figure 8:**
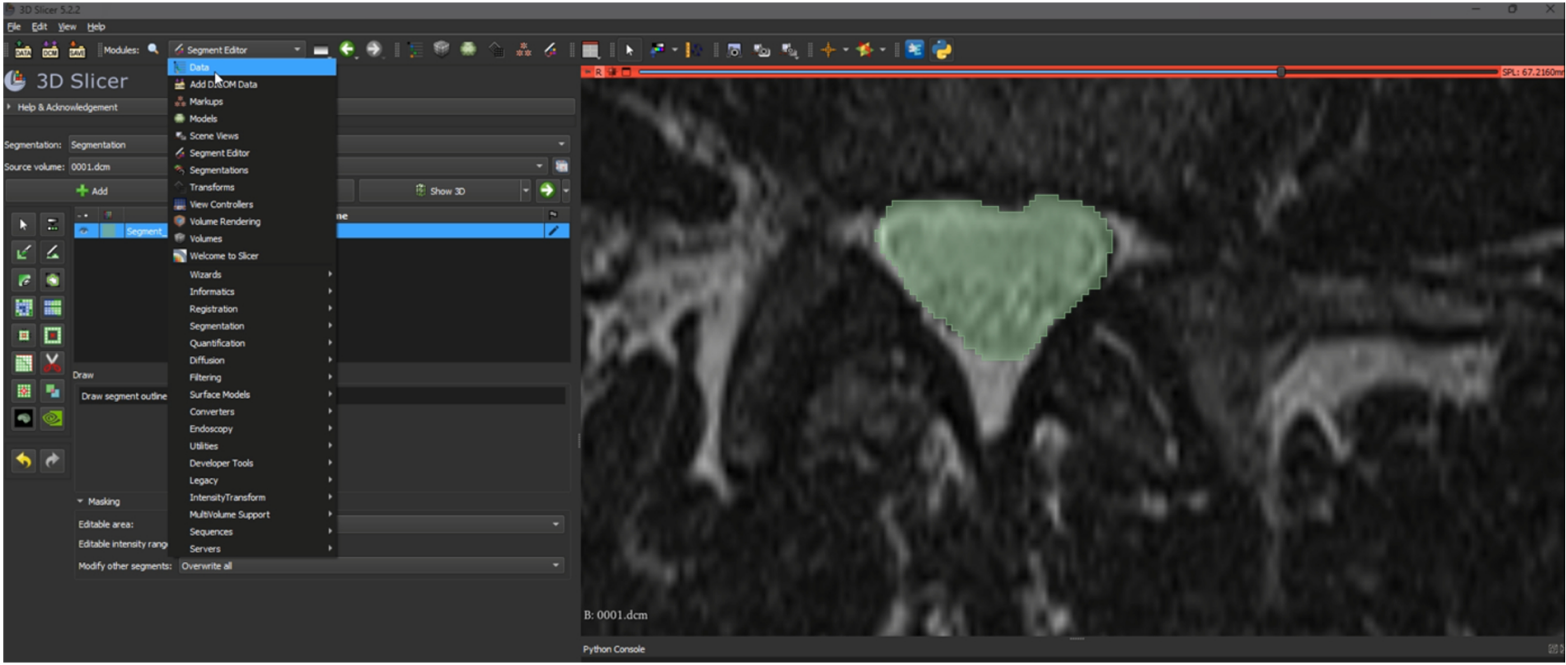
Scatter plot depicting the relationship between actual and predicted DSCA areas. Each point represents a single image from the validation set (identified using the best model from k-fold cross-validation with sklearn.model_selection in Python), showing results for the Attention U-Net model. Pearson's correlation coefficient: 0.9687.

**Figure 9:**
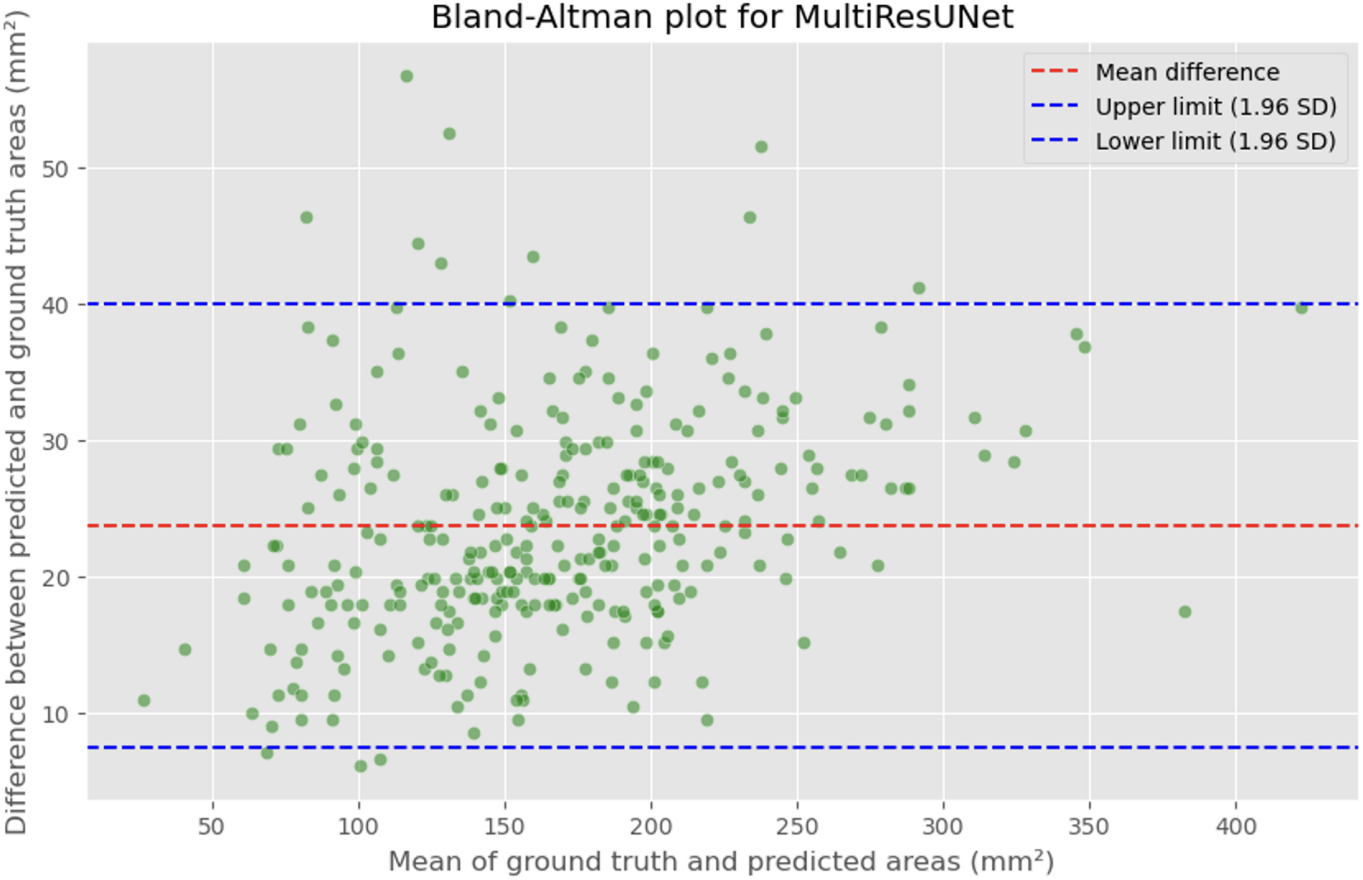
Bland-Altman plot showing the difference between predicted and actual DSCA areas against their mean. The red dashed line represents the mean difference, with values indicated for the MultiRes U-Net model. The blue dashed lines represent the limits of agreement, calculated as the mean difference ± 1.96 times the standard deviation of the differences. The mean difference is 23.7302'mm^2^, with limits of agreement ranging from 7.4189 to 39.9874'mm^2^.

**Figure 10:**
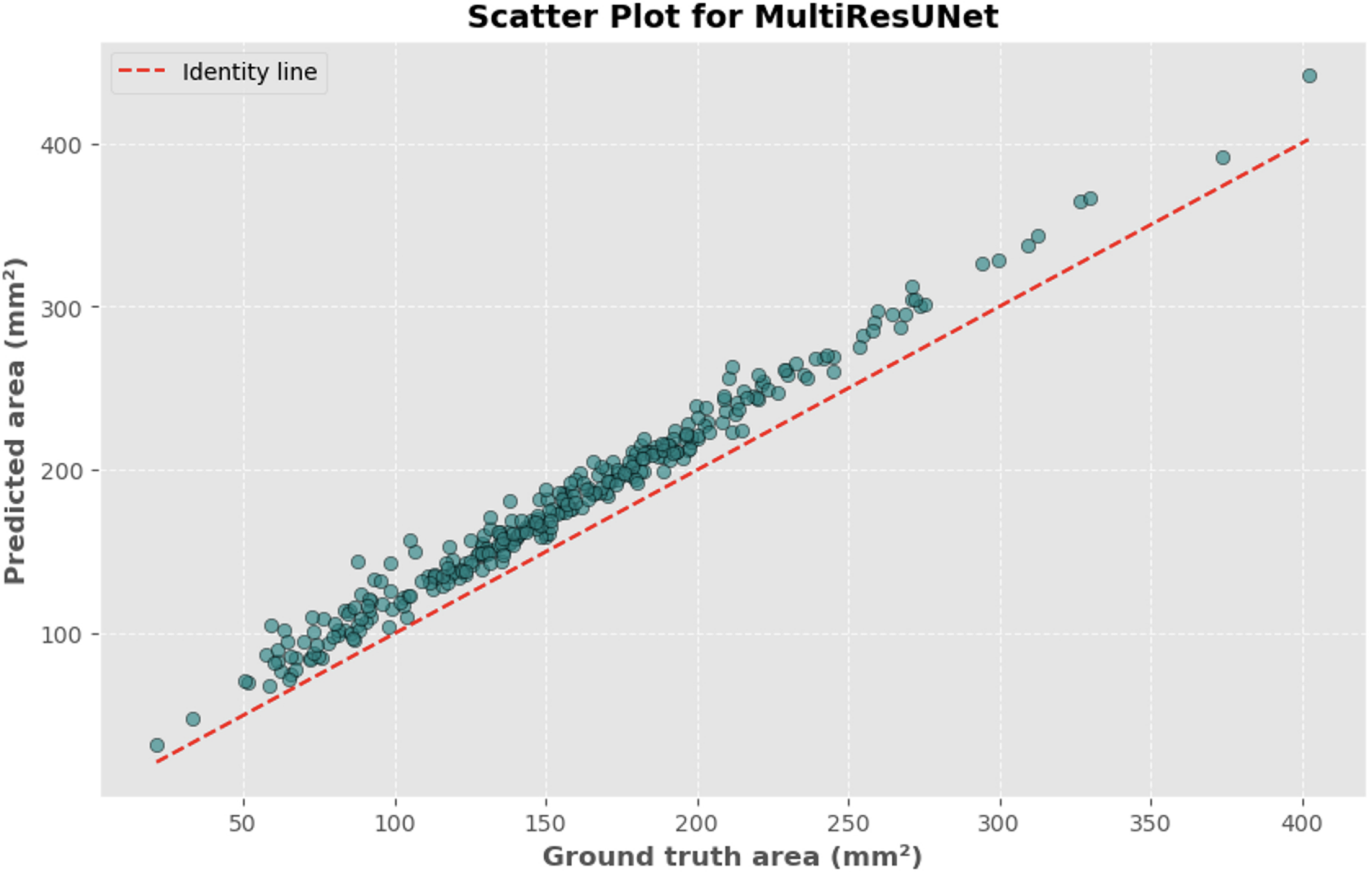
Scatter plot depicting the relationship between actual and predicted DSCA areas. Each point represents a single image from the validation set (identified using the best model from k-fold cross-validation with sklearn.model_selection in Python), showing results for the MultiRes U-Net model. Pearson's correlation coefficient: 0.9917.

The automated process of segmenting and calculating the dural sac cross-sectional area (DSCA) showed a strong correlation with the actual DSCA across all the models tested. Pearson correlation coefficients were observed to be 0.9323 for U-Net, 0.9687 for Attention U-Net, and 0.9917 for MultiResUNet. These coefficients reflect a very strong positive correlation between the predicted and actual areas, underscoring the high accuracy of the segmentation methods used (Figures 5–10). This demonstrates the models’ robustness and reliability, confirming their capability for accurate DSCA measurement. Among the models, the MultiResUNet exhibited the highest correlation coefficient, suggesting it may be more effective for DSCA segmentation and calculation in this context. Additionally, the MultiResUNet's lower Mean Absolute Error (MAE) and Mean Squared Error (MSE) values, both in initial and external validation phases, indicate its superior accuracy in predicting DSCA.

The MAE is a critical metric for evaluating model performance, representing the average absolute difference between predicted and actual areas, with lower MAE values indicating better model performance. The MAE values for our models were as follows: 23.7032 mm^2^ for MultiResUNet, 34.1307 mm^2^ for Attention U-Net, and 36.6760 mm^2^ for U-Net. Figures 4, 7, 9 present the Bland-Altman plots, with limits of agreement ranging from 7.4189 to 39.9874 mm^2^ for MultiResUNet, 4.2782 to 63.9832 mm^2^ for Attention U-Net, and −6.8582 to 79.6779 mm^2^ for U-Net.

The mean difference, indicating the average discrepancy between the predicted and actual DSCA values, was 36.4098 mm^2^ for U-Net, 34.1307 mm^2^ for Attention U-Net, and 23.7032 mm^2^ for MultiResUNet. These measurements, along with the limits of agreement describing the range within which most differences between predicted and actual DSCA lie, support the quantitative evaluation of model accuracy and the identification of patterns in prediction errors.

### 3.2 Accuracy analysis

In our study, three different convolutional network architectures—U-Net, Attention U-Net, and MultiResUNet—were evaluated for their accuracy using a dataset of 683 lumbar spine MRI scans. The evaluation was conducted using 5-fold cross-validation to ensure model robustness and generalizability. Additionally, external validation with 50 independent MRI scans was performed to verify the results (Table 1).

**Table 1:** Overview of training and validation accuracies of the three models across all folds of the cross-validation.

Model	Fold	Training accuracy	Validation accuracy
U-Net	1	0,997624	0,999017
U-Net	2	0,997956	0,998985
U-Net	3	0,997260	0,999294
U-Net	4	0,997764	0,999098
U-Net	5	0,997936	0,999141
U-Net	Average	0,997708	0,999107
Attention U-Net	1	0,996513	0,996771
Attention U-Net	2	0,996672	0,998759
Attention U-Net	3	0,997646	0,988654
Attention U-Net	4	0,997738	0,999182
Attention U-Net	5	0,996648	0,996893
Attention U-Net	Average	0,997043	0,996052
MultiResUNet	1	0,996392	0,999482
MultiResUNet	2	0,996659	0,999571
MultiResUNet	3	0,997004	0,999452
MultiResUNet	4	0,994499	0,999578
MultiResUNet	5	0,997246	0,999434
MultiResUNet	Average	0,996360	0,999503

The U-Net model exhibited impressive consistency in training accuracy, with values tightly clustered around a mean of 99.77%. Validation accuracy was similarly high, averaging 99.91%. The lowest validation accuracy was 99.90% in the first fold, while the highest was 99.93% in the third fold. The Attention U-Net, known for its ability to highlight relevant features in images, achieved an average training accuracy of 99.70% and an average validation accuracy of 99.61%. Although these figures were slightly lower than those of the U-Net model, the Attention U-Net showed remarkable stability across different folds, with a minimum validation accuracy of 98.87% in the third fold and a maximum of 99.92% in the fourth fold.

The MultiResUNet, featuring a hybrid architecture with multiple resolution paths to capture a variety of contextual information, demonstrated an average training accuracy of 99.64% and an exceptional validation accuracy of 99.95%. This underscores the efficiency of this architecture in processing medical images. Accuracy across individual folds was remarkably stable, with the lowest validation accuracy of 99.94% in the fifth fold.

In summary, all three models demonstrated high accuracy in both training and validation, confirming their suitability for analyzing lumbar spine MRI scans. However, the MultiResUNet stood out, particularly due to its validation accuracy.

To comprehensively assess model performance, several metrics were utilized, including accuracy, precision, recall, F1-score, and mean absolute error (MAE) (Table 2). The MAE for this model was 17.9487 mm^2^, highlighting its remarkable capability in estimating the dural sac cross-sectional area. The U-Net model achieved an accuracy of 0.9990, a recall of 0.9943, a precision of 0.7673, an F1-score of 0.8662, and an MAE of 46.5972 mm^2^.

**Table 2:** Comparative performance metrics of the three segmentation models (U-Net, attention U-Net, multiResUNet) evaluated using 5-fold cross-validation and external validation, including precision, recall, F1-score, and mean absolute error (MAE).

Model	Dataset	Precision	Recall	F1-Score	MAE (mm^2^)
U-Net	Main dataset	0.7673	0.9943	0.8662	46.5972
Attention U-Net	Main dataset	0.7966	0.9903	0.8829	39.4140
MultiResUNet	Main dataset	0.8966	0.9996	0.9453	17.9487
U-Net	External validation	0.7545	0.9912	0.8553	50.8731
Attention U-Net	External validation	0.7867	0.9871	0.8756	43.4768
MultiResUNet	External validation	0.8862	0.9989	0.9393	20.7329

When evaluated on the external validation dataset, the MultiResUNet model continued to show excellent performance, with an accuracy of 0.9995, a precision of 0.8862, a recall of 0.9989, and an F1-score of 0.9393. The MAE for this model was 20.7329 mm^2^, indicating a slight increase in error compared to the main dataset but still demonstrating very good performance. The Attention U-Net recorded an accuracy of 0.9989, a precision of 0.7867, a recall of 0.9871, an F1-score of 0.8756, with an MAE of 43.4768 mm^2^. The U-Net showed an accuracy of 0.9987, a precision of 0.7545, a recall of 0.9912, an F1-score of 0.8553, with an MAE of 50.8731 mm^2^. Despite these differences in performance, all models exhibited potential for clinical application with additional training on larger datasets.

**Figure 11:**
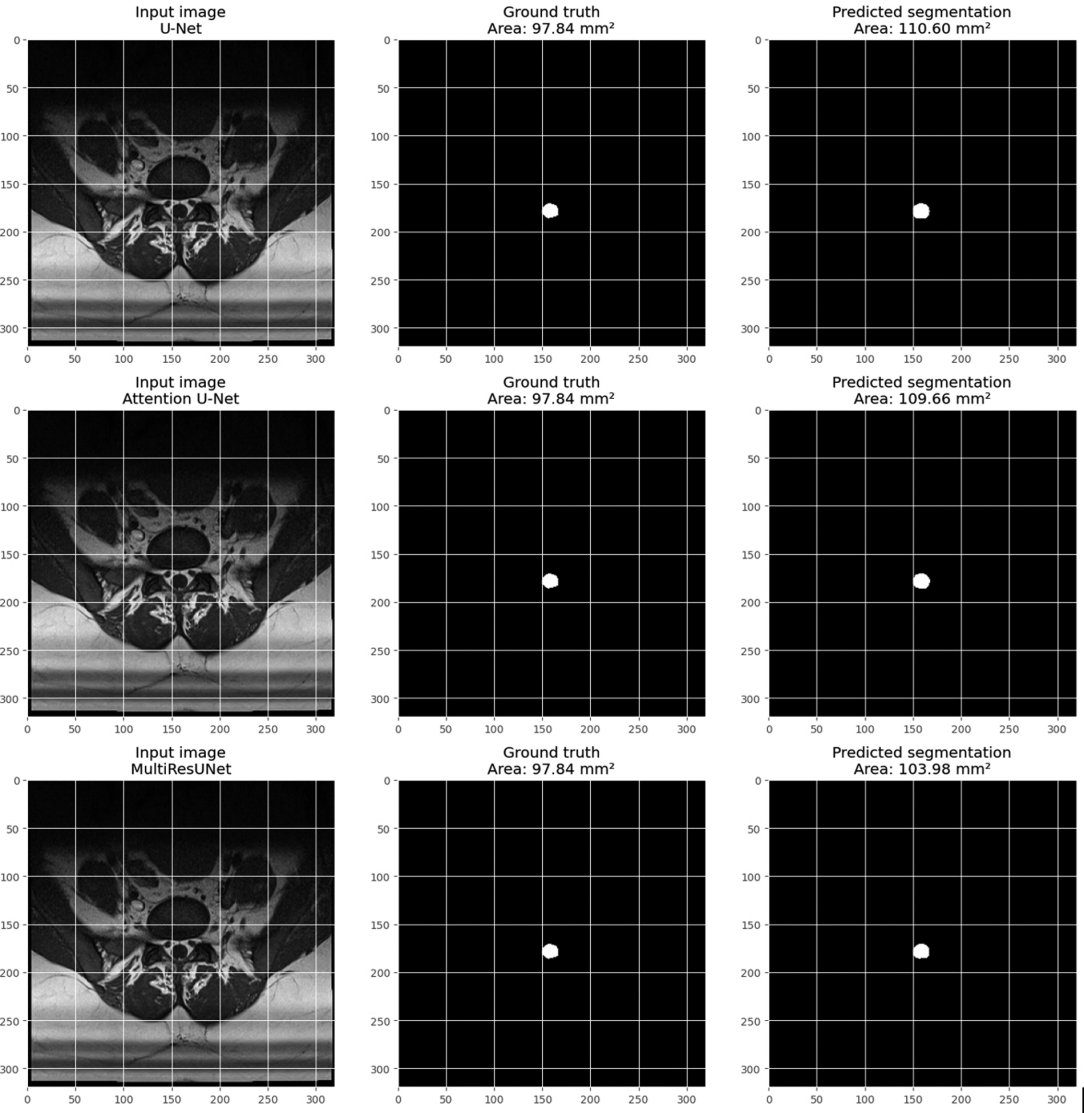
Visualization of an example from the main dataset (validation set, obtained through train_test_split from sklearn.model_selection in python) for each model (U-Net, attention U-Net, and multiResUNet). Each example includes three images: the input image, the Ground-Truth mask, and the predicted segmentation mask. The pixel size in millimeters is set based on the volume information in the main dataset. The DSCA is calculated for each predicted and Ground-Truth mask using the calculate_area() function. The calculated areas are displayed in the titles of the Ground-Truth and prediction images.

Figure 11 provides a visual example from the validation set for each model, including three images: the input image, the ground truth mask, and the predicted segmentation mask. The pixel size is set to 0.6875 millimeters, based on volume information from the main dataset. The calculate_area() function was used to determine the dural sac cross-sectional area for each predicted and actual mask, and the calculated areas are displayed in the titles of the actual and predicted images. These visualizations demonstrate the models’ effectiveness in segmenting the dural sac region in the input images, with the predicted segmentation masks closely matching the ground truth masks, particularly for the MultiResUNet, which showed a high level of agreement with the ground truth mask.

## 4 Discussion

This study focused on developing and evaluating deep learning models for the automated segmentation and quantification of the dural sac cross-sectional area (DSCA) in lumbar spine MRI images. Three different model architectures were examined: U-Net, Attention U-Net, and MultiResUNet. The aim was to assess the accuracy and efficiency of these models in DSCA measurement, contributing to the automation and enhancement of diagnostic processes in radiological practice.

The results demonstrate that all three models exhibited a high correlation between the predicted and actual DSCA values. The Pearson correlation coefficients were 0.9323 for the U-Net model, 0.9687 for the Attention U-Net, and 0.9917 for the MultiResUNet. This indicates a strong positive correlation, suggesting high segmentation accuracy. Notably, the MultiResUNet model achieved the best results, surpassing both the U-Net and Attention U-Net in terms of correlation. This implies that MultiResUNet is the most suitable for DSCA segmentation and calculation in this context. The Bland-Altman analysis and the calculation of the Mean Absolute Error (MAE) support these findings. The MultiResUNet model had the lowest MAE of 23.7032 mm^2^, followed by 34.1307 mm^2^ for the Attention U-Net and 36.6760 mm^2^ for the U-Net. These values underscore the superior performance of the MultiResUNet model in accurately predicting DSCA. The Bland-Altman plots showed that most differences between predicted and actual DSCA values were within the limits of agreement, further confirming the models’ robustness and reliability. In addition to the high correlation and low MAE, the MultiResUNet model also showed outstanding performance in accuracy analysis. It achieved an average training accuracy of 99.64% and a validation accuracy of 99.95%. These results exceeded those of the other two models, highlighting the effectiveness of MultiResUNet in image segmentation. External validation with an independent dataset of 50 MRI scans confirmed the generalizability of the models. The MultiResUNet model again demonstrated excellent accuracy of 99.95%, a high recall of 0.9989, and an F1-score of 0.9393. The MAE was 20.7329 mm^2^, indicating a slightly higher error rate compared to the main dataset but still showing very good performance. Overall, the developed deep learning models, particularly the MultiResUNet, exhibit high accuracy and reliability in the automated segmentation and quantification of DSCA in lumbar spine MRIs. These results highlight the potential of such models to support radiological practice, improving diagnostic accuracy and efficiency in assessing spinal pathologies. Future research should aim to further refine these models and apply them to larger and more diverse datasets to confirm and expand their clinical applicability.

The findings of this study underscore the high performance of deep learning models, especially MultiResUNet, in the automated segmentation and quantification of DSCA in lumbar spine MRI images. These results align with previous research highlighting the potential of deep learning methods for medical image analysis. Only a limited number of studies have investigated the performance of AI architectures in assessing spinal canal stenosis in MRI images. A study by Hallinan et al. evaluated a deep learning architecture for the qualitative assessment of spinal canal stenosis in four categories, ranging from normal to severe (25). Similarly, Bogdanovic et al. (2024) applied a deep learning algorithm to quantify lumbar spinal canal stenosis, finding high agreement between radiologists and the algorithm overall, which was interpreted as nearly perfect (26). Some further studies have also conducted qualitative assessments of spinal canal stenosis using AI architectures, with results comparable to Hallinan et al. or slightly worse (27, 28).

Recent studies have shown that U-Net-based deep learning models achieve remarkable accuracy and precision in DSCA measurement. The MultiResUNet demonstrated the best performance among the tested models, with an accuracy of 0.9996 in the main dataset and 0.9995 in the external validation dataset. This high accuracy and low MAE of 17.9487 mm^2^ and 20.7329 mm^2^, respectively, emphasize the superiority of this model compared to other tested architectures (29). A comparable study investigated the application of a 3D U-Net model for the automated segmentation of the dural sac in CT myelograms (CTM) of patients with lumbar spinal canal stenosis (LSS) (30). The results demonstrated high accuracy and generalizability of the developed model, with an average Dice coefficient (DCS) of 0.933 in the independent test and external validation datasets. These findings confirm the effectiveness of deep learning models in segmenting and quantitatively analyzing spinal structures. Similarly, Sanja et al. (2024) validated a fully automated AI model for quantitative measurements of the spinal canal in lumbar spine MRIs (26). Their retrospective study with 100 clinical patients showed that the AI model achieved human-level accuracy in measuring the anteroposterior (AP) and mediolateral (ML) diameters of the dural sac. The differences between the AI model and radiologists were minimal and likely within a clinically acceptable range (26). Our results align with those of Sanja et al., particularly regarding the high accuracy of AI models in segmenting and measuring DSCA. Both studies show that AI models can perform consistent and precise measurements comparable to human experts. In our study, the MultiResUNet model demonstrated remarkable performance with a mean absolute error (MAE) of 23.7032 mm^2^ in the main dataset and 20.7329 mm^2^ in the external validation dataset, underscoring the high accuracy and reliability of this model. An important aspect in Sanja et al.'s study was evaluating the AP and ML diameters of the dural sac (26). The average measurements by AI models and radiologists showed minimal differences, with mean absolute errors for the AP diameter ranging from 0.59 mm to 0.75 mm and for the ML diameter from 1.16 mm to 1.37 mm. These differences fell within a submillimeter range and were clinically insignificant (26). Similarly, our models achieved high correlations between predicted and actual DSCA values, further confirming the models’ robustness and accuracy. Regarding the measurement of the dural sac's cross-sectional area, Sanja et al.'s results showed a slight overestimation by the AI model compared to radiologists. This difference was about 13 mm^2^–15 mm^2^ and was deemed clinically acceptable (26). Our study also found overestimation, but with high accuracy in area measurement, and the MultiResUNet achieved the best results. The MAE for the main datasets was 17.9487 mm^2^, indicating high precision and reliability. Another crucial point in Sanja et al.'s study was the high inter-rater agreement in measuring the AP and ML diameters and the dural sac's cross-sectional area, demonstrated by high intraclass correlation coefficients (ICC) (26). These findings align with our observations that AI models can deliver consistent and reproducible measurements comparable to those of human experts. Sanja et al. emphasized the importance of the generalizability of their AI model by including data from various institutions and different MRI scanners (26). In our study, a validation dataset was also used to test the models’ generalizability, resulting in similarly positive outcomes.

A recently published study examined the performance of an AI architecture for evaluating various qualitative parameters in degenerative lumbar spine diseases using axial T2-weighted MRI images (31). This AI architecture, specifically trained for dural sac segmentation, achieved a Dice coefficient of 0.93 in evaluating an internal test dataset, which included 1.5T and 3T MRI scans from a single institution. Interestingly, our results closely match these findings. Our study demonstrates similar performance, with the MultiResUNet model achieving outstanding results. Compared to the mentioned studies, our model also achieved high accuracy values, underscoring the accuracy and reliability of dural sac segmentation. These results confirm the suitability of deep learning models for accurately segmenting medical images. A study from 2019 employed a similar methodology to ours, using a DCNN architecture with a U-Net model to segment axial T2-weighted MRI images of the lumbar spinal canal (32). In this study, the Dice coefficients for segmentations by the AI architecture and human readers ranged between 0.83 and 0.84. These values are significantly lower than those achieved by Bogdanovic et al. (26) and our accuracy analyses. Additionally, the Dice coefficient between the two human observers was 0.9, which is also lower than that observed by Bogdanovic et al. (26).

Our findings align with previous studies that have observed a tendency for AI-based segmentation models to slightly overestimate DSCA (29). While this overestimation may be small, it could potentially influence the clinical interpretation of borderline cases, where accurate measurements are crucial for determining the severity of spinal stenosis or the need for surgical intervention. For instance, a perceived increase in DSCA might prompt more conservative management when, in reality, more aggressive treatment could be warranted. However, the actual clinical impact of such discrepancies needs further investigation, including prospective studies that correlate measured DSCA values with patient outcomes and decision-making processes. Ultimately, clear communication of model limitations, along with the incorporation of additional clinical and imaging data, may help clinicians contextualize and appropriately integrate AI-based measurements into their diagnostic and treatment paradigms.

The application of AI-based segmentation in medical imaging, particularly for spinal pathologies, has seen significant advancements in recent years. While our study demonstrates the superior performance of the MultiResUNet model, recent works provide additional context to underscore its innovation and clinical relevance. A study by Isensee et al. revisited the nnU-Net architecture, emphasizing rigorous validation and adaptability across diverse medical imaging tasks, including volumetric spine imaging (17). While nnU-Net has achieved state-of-the-art results, its extensive computational requirements pose practical challenges, which our MultiResUNet addresses by delivering comparable accuracy with a more resource-efficient architecture. Similarly, studies like those by Yousef et al. on modified U-Net architectures for brain imaging segmentation highlight the importance of architectural innovations, such as attention mechanisms and multi-resolution analysis, in achieving precise results (14). Our MultiResUNet leverages these concepts, tailored specifically for the complexities of DSCA segmentation in lumbar MRI, showcasing its adaptability to spine-specific challenges. In the domain of lumbar spine imaging, Fan et al. introduced a deep learning model for CT myelogram segmentation, achieving Dice coefficients of 0.933. Although these results are impressive, they lack the external validation and diversity of imaging conditions included in our study (30). Moreover, our focus on T1-weighted MRI—a modality preferred for its superior soft tissue contrast—enhances the applicability of our findings to routine clinical workflows. Discrepancies in performance metrics across studies may arise from differences in imaging modalities, dataset characteristics, and segmentation goals. For example, while studies like Hallinan et al. focused on stenosis classification, our quantitative approach to DSCA measurement addresses a critical diagnostic need with greater precision (25). Furthermore, the inclusion of architectural innovations, such as attention mechanisms and multi-resolution paths, likely contributed to the MultiResUNet's ability to minimize mean absolute errors (MAE) compared to simpler architectures. Notably, emerging applications of deep learning in related domains, such as brain and cardiac imaging, further highlight the versatility of these technologies. For example, recent work on U-Net variants for cardiac segmentation demonstrated that multi-scale feature extraction significantly improved segmentation accuracy in complex anatomical regions (33). These insights reinforce the potential of our MultiResUNet model to extend beyond lumbar spine imaging, providing a scalable framework for other anatomical regions.

Our study demonstrates that our deep learning models, particularly the MultiResUNet, can perform accurate and reliable dural sac segmentations, matching or exceeding the best results reported in the literature. This highlights the importance of further developing and validating such models to optimize their application in clinical practice. Although several technical articles describe AI-based segmentations of the spinal canal and dural sac, few studies use large datasets. Our study contributes to closing this gap by providing detailed quantitative analyses and comparative investigations of multiple models. In summary, the deep learning models developed and validated in our study, especially the MultiResUNet, show high performance and accuracy in segmenting and measuring the dural sac's cross-sectional area in lumbar spine MRIs. These results align with findings from other recent studies, emphasizing these models’ potential to improve diagnostic accuracy and efficiency in clinical practice. Future studies should aim to further validate these models’ generalizability and explore their integration into clinical routines.

A critical step towards clinical adoption involves integrating the proposed solution seamlessly into existing radiology workflows, including Picture Archiving and Communication Systems (PACS) and electronic health record (EHR) infrastructures. Differences in MRI acquisition protocols, scanner types, and image quality across institutions may require site-specific calibration and periodic retraining to ensure consistent performance. Furthermore, clear interpretability of model outputs and user-friendly interfaces are necessary to build clinician trust and facilitate routine use. Addressing regulatory and compliance requirements, particularly regarding patient data privacy and device certification, will further influence the timeline and ease of clinical implementation. Finally, establishing robust training programs and providing clinical decision support tools will assist radiologists and clinicians in effectively incorporating automated quantification results into their diagnostic and treatment workflows.

Unlike previous studies, the present study used a larger data pool from two extensive online datasets (SPIDER MRI Dataset and Lumbar Spine MRI Dataset), utilizing the largest currently available dataset for automated DSCA measurement. A significant strength of the present study is the use of deep learning methods, which, unlike traditional semi-automated approaches such as those used by El Mendili et al. (20), offer higher scalability and accuracy. El Mendili et al. developed a semi-automated algorithm for segmenting the dural sac in cervical and thoracic MR images. Although they achieved high accuracy, the present study demonstrated that deep learning models, especially MultiResUNet, exhibit even higher precision and lower errors (20).

The present work shows that deep learning models can accurately measure DSCA and provide consistent and reproducible results, reducing variability between different observers and increasing efficiency in clinical practice. These results are particularly relevant as manual segmentation of the dural sac is time-consuming and can lead to inconsistent results. Fan et al. highlighted that automatic segmentation is a reliable method for quantifying DSCA, especially when assessing central and lateral LSS. This method could thus improve diagnostic accuracy and treatment planning by providing accurate and consistent measurements (30). Further development is needed to classify different stenosis types and forms using AI algorithms. Overall, the results of this study and the literature evidence show that deep learning models, particularly MultiResUNet, are a promising tool for automated DSCA measurement in lumbar spine MRIs. These models could improve diagnostic accuracy, reduce radiologists’ workload, and increase measurement consistency and objectivity. Future studies should focus on further validating these models with larger and more diverse datasets and exploring their integration into clinical practice.

### 4.1 Strengths and limitations

The study has several limitations. First, the models were trained and validated on a relatively limited dataset. To confirm the generalizability of the method, future research should utilize larger datasets from various institutions. Second, the method is currently restricted to calculating DSCA in T1-weighted axial MRI images of the lumbar spine. One key reason for focusing exclusively on T1-weighted axial images is the availability and consistency of these datasets, which ensured a stable training environment and reduced variability arising from differing acquisition parameters. T1-weighted images also provide robust anatomical context, capturing essential tissue boundaries that help delineate the dural sac's morphology. However, we acknowledge that T1-weighted scans may not exploit the high fluid contrast and signal intensities offered by T2-weighted sequences, potentially limiting the direct applicability of our model to other clinical contexts or MRI protocols that use alternative weightings. Incorporating T2-weighted images or multi-sequence data in future investigations could improve the generalizability of our approach, as well as enable domain adaptation techniques to extend the model's capabilities beyond the T1-weighted domain. Ultimately, expanding the training dataset to include various MRI contrast types will bolster the model's flexibility and utility across a wider range of clinical imaging environments. Another limitation is the lack of demographic data, which prevented a comprehensive analysis of additional study variables that might influence model performance. Future studies should include datasets with complete demographic information to provide insights into these effects. Additionally, this study focused exclusively on detecting and quantifying DSCA. Further research is needed to explore the clinical implications of using deep learning models for automated DSCA measurement in other anatomical structures. Future research should also include shape analysis alongside area measurements, as spinal canal morphology might be as important as canal area for stenosis assessment. Developing diagnostic shape measures will thus be a focus of future research.

Despite these limitations, our study makes a valuable contribution to research on deep learning applications in medical imaging. It shows that the presented U-Net-based models not only achieve high accuracy and reliability in DSCA measurement but also offer practical advantages that can enhance diagnostic processes in clinical practice. There are several strengths that underline its significance and relevance in medical imaging. A primary advantage of this study is the high accuracy and reliability demonstrated by the developed deep learning models, particularly the MultiResUNet, in the automated segmentation and quantification of the dural sac cross-sectional area (DSCA) in lumbar spine MRIs. This strong performance was evident in both the primary dataset and an external validation dataset, underscoring the robustness and clinical potential of these models. A key strength of our approach lies in the diversity of the patient datasets utilized, encompassing individuals with varying spine characteristics and clinical presentations, not limited solely to those with disc herniations. By including multiple representative slices per patient, we captured a wide range of dural sac anatomies and imaging conditions, enhancing the model's adaptability and robustness. This strategy, in turn, strengthens the model's potential for generalization, increasing its clinical utility and facilitating its application across diverse patient populations. Automating DSCA measurement could significantly reduce radiologists’ workload and improve diagnostic efficiency, a critical need given the aging population and increasing demand for diagnostic imaging studies. Additionally, the study's focus on DSCA measurement in T1-weighted axial MRI images of the lumbar spine offers several benefits. These images provide high anatomical detail and are less prone to artifacts compared to T2-weighted images. The findings suggest that U-Net-based deep learning models can achieve high accuracy and recall, indicating their suitability for clinical applications. This study also adds to the broader conversation about the application of deep learning models in medical image analysis, confirming the versatility of U-Net architectures, which have been successfully used in various tasks such as cell segmentation, organ segmentation, and tumor detection.

The clinical significance of automated dural sac area measurement in lumbar MRI extends beyond mere technical accuracy; it directly impacts how clinicians assess spinal canal stenosis, plan interventions, and monitor disease progression. Precise and reproducible measurement of the DSCA is commonly used to help determine the severity of conditions such as lumbar spinal canal stenosis, which remains a leading cause of lower back pain and neurological deficits. Traditional manual or semi-automatic measurement methods can be time-consuming and subject to inter- and intra-observer variability, impeding efficient workflow in busy clinical settings. By contrast, our deep learning–based models offer a rapid, reliable, and fully automated approach, thereby reducing the burden on radiologists and enabling more consistent evaluations across different clinical sites. Furthermore, automated DSCA measurement has potential to assist in risk stratification and disease progression monitoring. For example, smaller cross-sectional areas have been linked to more pronounced neurological symptoms and may serve as an indicator of surgical necessity. Integrating the proposed solution into existing clinical workflows could enhance decision-making by alerting clinicians to borderline or high-risk patients who could benefit from earlier or more aggressive intervention. Moreover, longitudinal tracking of DSCA through automated solutions can help clinicians assess the effectiveness of treatments—such as physical therapy, epidural injections, or surgical decompression—over time. This aligns well with standardized clinical guidelines that emphasize the importance of quantitative imaging markers, but which currently rely heavily on subjective assessments or inconsistent manual measurements. When compared to existing clinical guidelines or standardized evaluation tools, our approach provides a novel paradigm by leveraging end-to-end convolutional networks to segment the dural sac in a fully automated fashion. This reduces subjective bias, while the high DSC and low MAE suggest that the predicted segmentations closely approximate the ground truth. These strengths highlight the innovation of our framework, which can be further expanded by integrating patient-specific parameters—such as demographic data and comorbidities—to improve predictive modeling and personalized care pathways. Hence, our study not only demonstrates technological feasibility but also paves the way for improved clinical management of lumbar spine disorders, bridging the gap between cutting-edge AI research and real-world patient benefit.

## 5 Conclusion

This research focused on developing and evaluating deep learning models for the automated segmentation and quantification of the dural sac cross-sectional area (DSCA) in lumbar spine MRI images. The findings indicated that the U-Net, Attention U-Net, and particularly the MultiResUNet models, demonstrated high accuracy and reliability in measuring DSCA. These models have the potential to enhance diagnostic accuracy and alleviate the workload of radiologists, which is increasingly critical in light of an aging population and the rising demand for diagnostic imaging. Among the models, the MultiResUNet showed the best performance, achieving a Pearson correlation coefficient of 0.9917 and a mean absolute error (MAE) of 23.7032 mm^2^ in the primary dataset. This high precision and reliability confirm the MultiResUNet model's potential for clinical application. External validation with an independent dataset of 50 MRI scans confirmed the models’ generalizability, with the MultiResUNet again showing outstanding results. These findings align with other recent studies and highlight the versatility and effectiveness of deep learning models in medical image analysis.

However, our study also has some limitations that must be considered. The models were trained and validated on a limited dataset, and the generalizability of our method must be confirmed using larger and more diverse datasets from different institutions. The calculation of DSCA was restricted to T1-weighted axial MRI images of the lumbar spine, although T2-weighted images might provide a clearer delineation of the dural sac. Future work should incorporate T2-weighted images to further improve accuracy. Additionally, the absence of demographic data limited our analysis, preventing a comprehensive evaluation of the impact of demographic variables on model performance. Future studies should use datasets with complete demographic information and investigate the potential clinical implications of automated DSCA measurement for other anatomical structures. Future research should also include shape analysis alongside area measurements, as spinal canal morphology may provide important diagnostic information. Despite these limitations, our study makes a valuable contribution to research on deep learning applications in medical imaging. The presented U-Net-based models, particularly the MultiResUNet, show high accuracy and reliability in DSCA measurement and offer practical advantages that can improve diagnostic processes in clinical practice. These results encourage further exploration and integration of such models into clinical routines to optimize diagnostic and treatment planning efficiency and accuracy.

## Data Availability

The original contributions presented in the study are included in the article/Supplementary Material, further inquiries can be directed to the corresponding authors.
