# Supplementary material for: Deep learning-based automated segmentation and quantification of the dural sac cross-sectional area in lumbar spine MRI
Source: Front Radiol. 2025 Mar 25;5:1503625. doi: 10.3389/fradi.2025.1503625 (PMC11975661; doi:10.3389/fradi.2025.1503625)
Supplement: Supplementary file 1 [file Table1.docx]

Supplementary Table 1: Contrasting the key architectural and functional differences between U-Net, Attention U-Net, and MultiResUNet.

| **Feature** | **U-Net** | **Attention U-Net** | **MultiResUNet** |
| --- | --- | --- | --- |
| Core Architecture | Standard encoder-decoder with skip connections | U-Net architecture enhanced with attention gates in skip connections | U-Net-based design incorporating multi-resolution blocks and residual pathways |
| Key Innovation | Symmetric structure and direct skip connections for feature reuse | Attention mechanisms that highlight relevant spatial regions and suppress irrelevant background | Multi-resolution analysis to capture features at various scales, leveraging ResPaths for more efficient feature propagation |
| Parameter Complexity | Generally fewer parameters, relatively simple design | Slightly increased parameter count due to attention gates | Similar or slightly higher complexity than U-Net due to multi-resolution blocks and additional convolutions |
| Focus of Improvement | Baseline for segmentation tasks, good general performance | Enhanced focus on regions of interest, improved segmentation in challenging areas | Better representation of features at multiple scales, potentially improving accuracy and robustness |
| Typical Applications | Broad range of medical imaging tasks, initial baseline model | Tasks where delineating subtle or small anatomical structures is critical | Complex imaging scenarios requiring robust feature extraction and aggregation across scales |
| Performance Trade-offs | Fast and efficient, may struggle with very subtle boundaries | Slightly more computationally intensive, but often yields more precise segmentations | Increased complexity may require more compute resources, but can achieve higher accuracy and stability in challenging datasets |
